# HEALTH-RELATED QUALITY OF LIFE AND CARDIOVASCULAR DISEASE IN ASIAN AMERICAN AND PACIFIC ISLANDER OLDER ADULTS

**DOI:** 10.1093/geroni/igz038.1902

**Published:** 2019-11-08

**Authors:** Lan Doan, Yumie Takata, Karen Hooker, Carolyn Mendez-Luck, and Veronica L Irvin

**Affiliations:** Oregon State University, Corvallis, Oregon, United States

## Abstract

Cardiovascular disease (CVD) is the leading cause of death for Asian American (AA), Native Hawaiian, and Pacific Islander (NHPI) older adults, and AAs/NHPIs have not enjoyed decreases in CVD mortality rates, as have non-Hispanic whites (NHWs). Heterogeneity exists in the prevalence of traditional CVD risk factors for AAs/NHPIs. Health-related quality of life (HRQOL) reflect physical and mental burdens beyond clinical burdens, which may help explain discrepant CVD rates and risk factors in AAs/NHPIs. We examined HRQOL among NHW and AA/NHPI Medicare Advantage enrollees with and without a CVD (i.e., coronary artery disease, congestive heart failure, myocardial infarction, and stroke) using the Medicare Health Outcomes Survey. The sample included 655,914 older adults who were 65 years or older, self-reported as AA/NHPI or NHW, and were enrolled in Medicare Advantage plans in 2011-2015. HRQOL was measured using the Veterans RAND 12-item survey and is composed of a physical component score (PCS) and mental component score (MCS), where higher scores reflect better physical and mental health, respectively. Multivariable linear regression was used to explore HRQOL and CVD prevalence. Asian Indian, Filipino, Vietnamese, Other Asian, and NHPI subgroups had lower overall PCS, and all AA/NHPI subgroups had lower overall MCS, compared to NHWs. Among those reporting having any CVD, PCS varied by CVD outcomes and subgroups, whereas MCS was lower for all CVD outcomes and for all but one AA/NHPI subgroups (Japanese), compared to NHWs. Attention to mental health for AA/NHPI older adults could be important for the equitable realization of healthy aging.

